# Synthesis of glycyrrhetinic acid-modified liposomes to deliver Murrayafoline A for treatment of hepatocellular carcinoma

**DOI:** 10.1007/s10856-022-06692-1

**Published:** 2022-10-04

**Authors:** Cuc Thi Dinh, Ha Thi Vu, Quynh Thi Huong Phan, Linh Phuong Nguyen, Toan Quoc Tran, Dung Van Tran, Nguyen Ngoc Quy, Dung Thuy Nguyen Pham, Duong Thanh Nguyen

**Affiliations:** 1 Institute of Chemistry, Vietnam Academy of Science and Technology (VAST), 18 Hoang Quoc Viet St., Cau Giay Dist., Hanoi, 10000 Vietnam; 2 Institute of Natural Products Chemistry, 18 Hoang Quoc Viet st., Cau Giay dist., Hanoi, 10000 Vietnam; 3 Hanoi Medical University, 1 Ton That Tung St., Dong Da Dist., Hanoi, 10000 Vietnam; 4 Graduate University of Science and Technology, 18 Hoang Quoc Viet st., Cau Giay dist., Hanoi, 10000 Vietnam; 5 VIET ANH VENTURE INVESTMENT J.S. COMPANY USA SANFORDPHARMA FACTORY, Hanoi, 10000 Vietnam; 6 Institute of Applied Technology and Sustainable Development, Nguyen Tat Thanh University, Ho Chi Minh City, 700000 Vietnam

## Abstract

Hepatocellular carcinoma is a common type of cancer associated with a high mortality rate. Among several bioactive compounds, Murrayafoline A (MuA) has been proved as a bio substance that exhibits great potentials in treating liver cancer. In order to overcome the high cytotoxicity and low solubility of MuA, a delivery system based on nanocarriers is necessary to deliver MuA towards the desired target. In the present study, 18β-glycyrrhetinic acid (GA), which is known as a ligand for liver targeting, was used to construct the cholesterol-poly (ethylene glycol)-glycyrrhetinic acid (GA-PEG-Chol) conjugate and liposome for MuA administration. The compound was then examined for therapeutic efficacy and safety in HUVEC and HepG2 cells in 2D and 3D cell cultures. Results have shown that MuA-loaded liposomes had IC_50_ value of 2 µM in HepG2 and had the cytosolic absorption of 8.83 ± 0.97 ng/10^5^ cells, while The IC50 value of MuA-loaded liposomes in HUVEC cell lines was 15 µM and the the cytosolic absorption was recorded as 3.62 ± 0.61 cells. The drug test on the 3D cancer sphere platform of the HepG2 cancer sphere showed that MuA-loaded GA liposomes had the highest efficacy at a concentration of 100 µg/mL. In short, these results suggest that MuA-loaded GA liposomes have the potential for maintenance drug delivery and liver targeting.

## Introduction

Cancer is recognized as the world’s leading cause of death, with approximately 10 million deaths in 2020 [1–3]. In particular, hepatocellular carcinoma is the most common type of primary liver cancer with an estimated 841,000 new cases and 782,000 deaths per year [4, 5]. This type of cancer often occurs in patients with chronic liver diseases, such as cirrhosis caused by hepatitis B or hepatitis C infection or the excessive alcohol consumption [6]. Currently, among several methods which have been developed for liver cancer treatment, including surgery, radiotherapy, interventional radiology, gastroenterology procedures, and immune therapy, chemotherapy has remained the most effective method for patients [3, 7]. Therefore, finding new potential drugs is necessary and urgent for the treatment of hepatocellular carcinomaas well as mortality rate reduction.

Murrayafoline A (MuA) is a plant alkaloid isolated from the root of several species of the genus *Clausena (Rutaceae)* and *Murraya* [8, 9]. This compound has many biological activities such as anti-cell growth (SW480, HCT116, MoLT-4 (acute lymphoblastic leukemia), HOP-18 (lung cancer) and LS174T) and anti-inflammator [10–14]. However, MuA has several disadvantages that can limit its efficiency and application in cancer treatment. For instance, the relatatively poor aqueous solubility and high cytotoxicity of MuA is can limit the dose usages and accidentally destroy other normal cells, such as bone marrow, hair follicles and epithelial cells in the mouth, digestive tract and reproductive system [12–14]. Therefore, it is necessary to develop a carrier for MuA delivery to enhance treatment efficacy.

Liposomes are sphere-shaped vesicles with an aqueous core surrounded by lipid bilayers [15]. They have been widely used as the drug carrier for improving efficacy and therapeutic index of the drug, increasing stability via encapsulation of non-toxic, flexible, biocompatible, completely biodegradable, nonimmunogenic materials for systemic and non-systemic administrations, and reducing the exposure of sensitive tissues [15–17]. Currently, many liposomal drugs have been approved by the FDA, such as Myocet to treat breast cancer [18], Doxil to treat ovarian cancer [19], and Marqibo to treat acute lymphoblastic leukemia [7]. Therefore, the liposome can be used for the delivery of MuA to protect the structure of the drug from metabolic degradation, mediate the dispersion of drugs in tissues, and increase absorption in organs.

In order to improve the accuracy of drug delivery towards tumors and organs, liposomes can be functionalized with various targeting ligands, such as antibodies, peptides or proteins, and small molecules. As compared to non-ligand liposomes, ligand-directed liposomes demonstrate special drug delivery in which the ligands can attach to a specific target such as receptors and antigens of the target cells with enhanced affinity and facilitate receptor-mediated endocytosis [20, 21]. To target the liver tissues, various ligands such as asialofetuin, fucose, galactose, lactobionic acid, mannose, mannose-6-phosphate (M6P), and peptides have been utilized to generate drug targeting moieties for the particular cells [22]. These target-specific binding ligandsdiscovered in all resident intrahepatic cells which are determined in various liver diseases such as Kupffer cells and HSCs [23–25]. 18*β*-glycyrrhetinic acid (GA, 3*β*-hydroxyl-11-oxo-olean-12-ene-29-oic acid, **1**), which is one of the main compounds extracted from *Glycyrrhiza glabra L*., was found to be capable of inhibiting liver carcinogenesis and cell proliferation of the human HCC cell line HepG2 [26–29]. In detail, the target binding site of GA - protein kinase C (PKC)- is more active in HCC than in other non-tumor liver cells. Sun *et al*. (2018) reported that GA demonstrates a targeted impact on HCC cells and tumors [30]. The most targeting effect is provided by the β-configuration hydrogen atom at the C_18_ location of GA. In addition, the C_11_-carbonyl and C_3_-hydroxy groups of GA display specific and minor effects on HCC targeting. According to Singh et al. (2018) [31], the use of GA-conjugation in diagnosis and therapies significantly improved their HCC targeting capacity and treatment efficiency. Therefore, it is assumed that through the interaction between GA and the over-expressed PKC, GA-modified liposomes could target selective HCC cells.

**Fig. 1:**
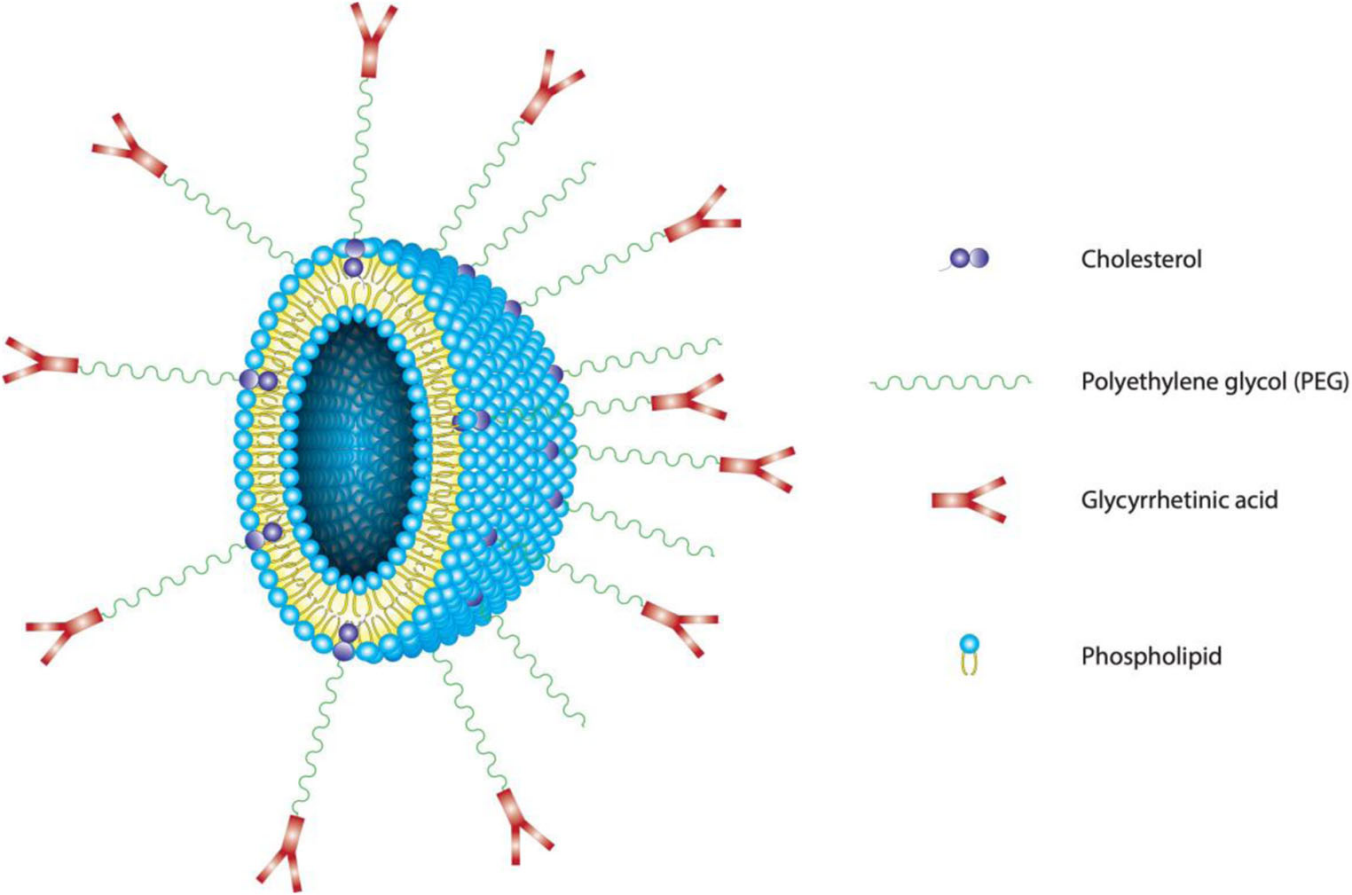
A multifunctional liposome with hydrophilic shell of PEG and glycyrrhetinic acid ligand

In this study, we developed GA-conjugated liposomes to deliver MuA for liver targeting (Fig. 1). Furthermore, polyethylene glycol (PEG) was utilized to increase circulation time and avoid eliminating the reticuloendothelial system (RES). With the hydrophilic chain shielding the surface of the liposome, PEG could suppress the positive charges and weaken the toxic response of non-specific electrostatic interactions of liposomes with normal cells. Our drug delivery system was tested on a three-dimensional (3D) cell culture model, which mimicked the in vivo platform for reproducibility under the physiological conditions.

## Materials and methods

### Materials

Glycyrrhetinic acid (GA), Poly(ethylene) glycol 2000 (HO-PEG_2000_–OH with 2 kDa of PEG chains), *N,N*′-Dicyclohexylcarbodiimide (DCC), Dichloromethane (DCM), 4-(Dimethylamino) pyridine (DMAP), anhydrous sodium sulfate (Na_2_SO_4_), succinic anhydride, 3-(4,5-dimethylthiazol-2-yl)-2,5-diphenyltetrazolium bromide (MTT) were purchased from Sigma-Aldrich (St.Louis, MO, USA). Cholesterol (Chol) was obtained from Avanti® Polar Lipids (Alabaster, AL, USA). Murrayafoline A (MuA) was extracted and isolated from the roots of *Glycosmis stenocarpa*. All other reagents were of analytical grade.

Structural analysis: NMR spectra of Chol-suc, Chol-PEG, Acetyl glycyrrhetinic acid, GA-PEG-Chol have been recorded on a Bruker Avance (500 MHz) spectrophotometer (Bruker Corporation, Massachusetts, USA) (CDCl_3_ as a solvent).

### Methods

#### Synthesis of GA-PEG-Chol conjugates

**Scheme 1:**
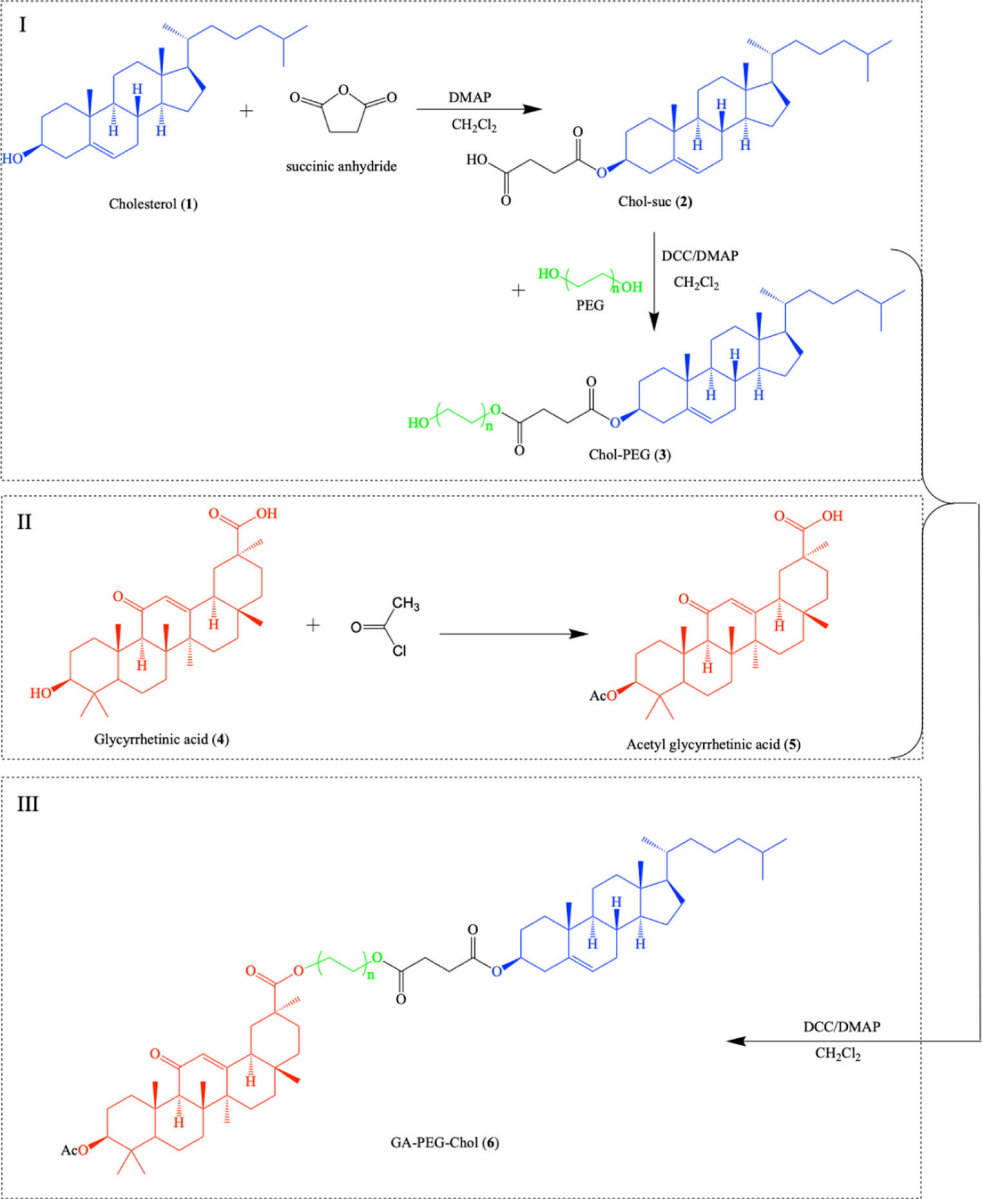
Chemical reactions for preparing the GA–PEG–Chol conjugates

The cholesterol-poly(ethylene)glycol-glycyrrhetinic acid (GA-PEG-Chol) conjugates were synthesized from cholesterol and glycyrrhetinic acid by 3 steps. The synthesis process of GA–PEG–Chol conjugates was displayed in Scheme 1.

**Fig. 2:**
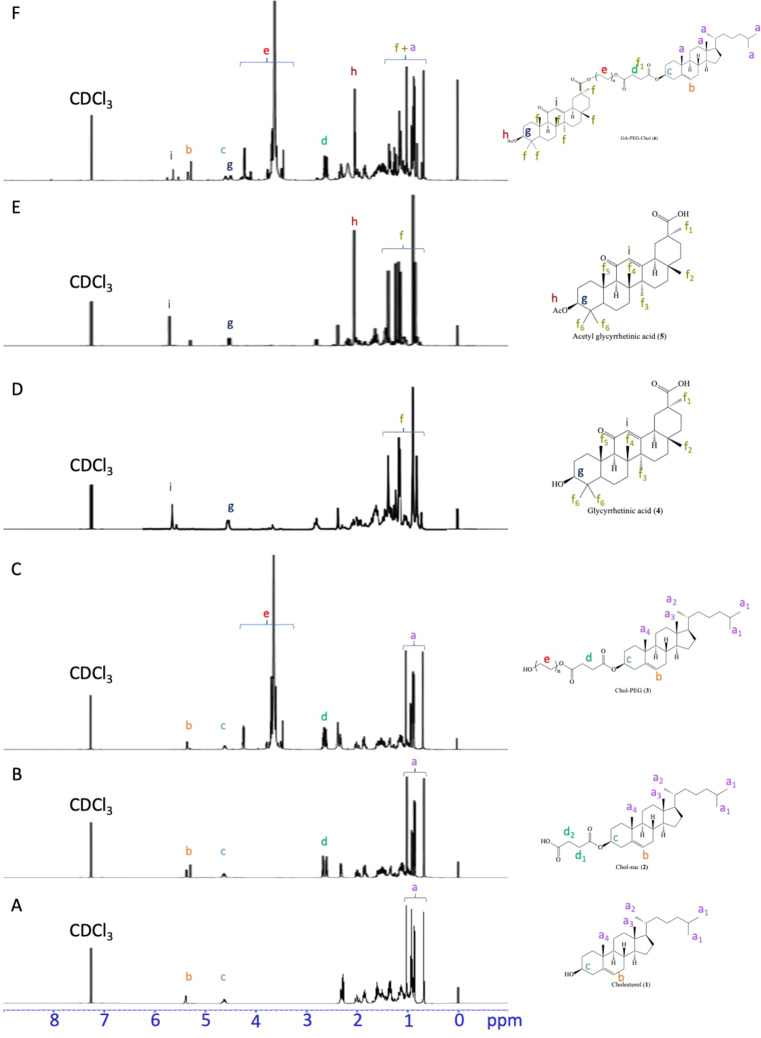
The ^1^H NMR spectra of (**A**) Cholesterol (**1**), (**B**) Chol-suc (**2**), (**C**) Chol-PEG (**3**), (**D**) GA (**4**), (**E**) acetyl glycyrrhetinic acid (**5**), and (F) GA-PEG-Chol (**6**)

(i) Synthesis of Chol-PEG (**3**): Cholesterol (Compound **1**, 1 mmol), succinic anhydride (2.25 mmol) in dry dichloromethane (DCM, 5 mL) were added to 4-dimethylamino pyridine (DMAP, 0.16 mmol) and pyridine (0.5 mmol). The reaction mixture was stirred under argon and refluxed for 3 days. Then, the mixture was cooled to room temperature before adding water. The solution was adjusted to pH 2–3 by using HCl 5%. Then, the reaction mixture was extracted with ethyl acetate (EtOAc, 20 ml x 3 times). The extract was dried with anhydrous Na_2_SO_4_, the removal solvent was *in vacuo*, and the cholesterol hemisuccinate (Chol-suc, compound **2**, yield 65%) was purified using a column on silica gel n-hexane/ethyl acetate (3:1). Then, Chol-suc (**2**, 1 mmol) and poly(ethylene)glycol 2000 (PEG-2000, 1.2 mmol) in DCM (5 mL) were added with DCC (1.5 mmol) and DMAP (0.5 mmol). The mixture was stirred for 48 h at room temperature, then DCM was removed under reduced pressure. The mixture was added to water and filtered, washed, dried at 70 ^o^C to obtain the white solid, yielding 85.4% (Chol-PEG, compound **3**). ^1^H-NMR (CDCl_3_, ppm) was shown in Fig. 2A–C.

(ii) Synthesis of acetyl glycyrrhetinic acid (**5**): GA (1 mmol) was dissolved in DCM (5 mL) and stirred at 0 ^o^C in an ice bath. Then, acetyl chloride (1.5 mmol) was added dropwise into the solution. The mixture was stirred for 10 h at room temperature, then the solvent was evaporated *in vacuo* and washed with water (50 mL x 2), dried at 70 ^o^C to provide white solid, yielding 78.6% (acetyl glycyrrhetinic acid, compound **5**). The dried product can be used in the next step. ^1^H-NMR (CDCl_3_, ppm) was shown in Fig. 2D.

(iii) Synthesis of GA-PEG-Chol (**6**): Compound **3** (1 mmol) in DCM (5 mL) was added with compound **5** (1 mmol), DCC (0.8 mmol), and DMAP (0.2 mmol), then stirred for 48 h at room temperature. Afterward, the solvent was removed under reduced pressure. The product was purified by column chromatography on silica gel using dichloromethane/methanol (10:1) to give GA-PEG-Chol, yielding 65% (compound **6)**. ^1^H-NMR (CDCl_3_, ppm) was shown in Fig. 2E.

#### Preparation of MuA-loaded liposome

Liposomes were synthesized by the film hydration method followed by extrusion. 0.01 M stock Chol-PEG-GA solution (27.6 μL), 250 μL of stock DOPC, and 109.3 μL of stock MuA were combined in a 1 mL Eppendorf. The mixture was vortexed for 60 s, then transferred to a bottom flask. Rotary evaporation was used to remove CHCl_3_. Distilled water (3 mL) was added and sonicated in a water bath at room temperature for 30 s. The resulted opaque suspension contained both MuA-encapsulated liposomes, and unencapsulated MuA. The liposome suspension obtained from thin-film hydration was dialyzed to remove the unencapsulated MuA. The suspension was dialyzed with 2 L distilled water for 24 h. Then, the suspension through the polycarbonate membrane by a mini-extruder. The samples were stored at 37 ^o^C or room temperature (25 ^o^C) when extrusion was finished. Blank liposomes were prepared in the same method without adding MuA.

#### Characterization of MuA-loaded liposome

The z-average, mean size, zeta potential and polydispersity index (PDI) of liposome samples stored at room temperature were determined by dynamic light scattering (DLS, Horiba, Japan). To assess the encapsulation efficiency (EE) and drug loading content (DL), water was first removed from the liposome solution using rotary evaporation. The liposome film was dissolved in 3.5 mL CHCl_3_/ MeOH (1:3, v/v) and bath sonicated for 2 min to release the entrapped MuA. The resulting solution was then diluted using 0.0029 L of CHCl_3_/MeOH [1:3, (v/v)]. The absorbance of the sample was measured at 243 nm using a UV-Vis spectrophotometer (Hitachi U-2900, Hitachi, Tokyo, Japan) against a standard of CHCl_3_/ MeOH (1:3, v/v). The EE% and DL% were calculated using the following equation:

$${{{\mathrm{EE}}}}\left( {{{\mathrm{\% }}}} \right) = \frac{{The\,amount\,of\,MuA\,in\,the\,liposomes}}{{The\,total\,amount\,of\,MuA}}x100\left( {{{\mathrm{\% }}}} \right)$$

$${{{\mathrm{DL}}}}\left( {{{\mathrm{\% }}}} \right) = \frac{{Mass\,of\,MuA\,in\,the\,liposomes}}{{mass\,of\,MuA/liposomes}}x100\left( {{{\mathrm{\% }}}} \right)$$

The samples were studied for 24 h to determine their release patterns and storage stability. Aliquots were removed at various time intervals to evaluate the size and EE. Dialysis bags (MWCO = 14,000 Da) were incubated in 50 mL PBS (pH 7.4 or pH 5.5) containing Tween 80 (0.5%, w/w) at 40 ^o^C with moderate shaking (100 rpm). At various time intervals, 1 mL of dialysis medium was collected and replaced with an equivalent volume of fresh PBS buffer. The results were the average values of three replicates and shown as mean ± SD.

#### In vitro cellular uptake and cell viability assay

##### Cell culture

Human hepatic cell line (HepG2) and human umbilical vein endothelial cells (HUVEC) were obtained from American Type Culture Collection (ATCC, Manassas, VA, USA).

In DMEM medium supplemented with 10% FBS (Invitrogen, Carlsbad, CA), 100 IU/mL penicillin, L-glutamine (2 mM), and 100 mg/mL streptomycin (Mediatech, Inc., USA), HepG2 cells were grown in a single layer. The cells were kept at 37 ^o^C in a humidified incubator under a 5% CO_2_ environment. Cells at the exponential phase of growth were used in the tests.

HUVECs were developed and kept in endothelial cell medium, EGM-2 BulletKit (Lonza, Basel, Switzerland), which contained EBM-2 medium (serum-free) supplemented with 10% FBS, human vascular endothelial cell growth factor (hVEGF), human fibroblast growth factor-B (hFGF-B), human epidermal growth factor (hEGF), long R insulin-like growth factor-1 (R3-IGF-1), hydrocortisone, heparin, and ascorbic acid. All HUVECs used in these investigations were cultivated and maintained in EGM-2 media in a humidified incubator (95% air and 5% CO_2_) at 37 ^o^C.

##### In vitro cytotoxicity

The MTT test was performed to determine the cytotoxicity of free MuA and different MuA-encapsulated liposomes. In a 96-well plate (Corning Inc., NY, USA), cells were seeded at a density of 1 × 10^4^ cells per well and incubated for 1 day. The medium was replenished after 24 h with MuA-loaded GA liposomes, MuA-loaded liposomes and free MuA solution at equivalent drug dosages ranging from 0.01 g/mL to 40 g/mL. After incubation, 2 mL of MTT reagent (5 mg/mL) was added to each well, and the cells were cultured for another 4 h at 37 ^o^C. Afterwards, the plate was covered in foil and stirred on an orbital shaker until the formazan was completely dissolved. The absorbance of each well was measured in 1 hour using a Spark® Plate Reader (Tecan, Switzerland) at OD = 590 nm.

$$Cell\,viability\left( {{{\mathrm{\% }}}} \right) = \frac{{A_{sample} - A_{blank}}}{{A_{control} - A_{blank}}}x100$$

#### Synthesis of cancer spheroid

Cytotoxicity of MuA-loaded liposomes was tested by using 3D cell culture. Cancer spheroids were created as described previously [32]. The photolithography technique was used for hydrogel-based microwells prepared from PEGDA. The solvent was phosphate-buffered saline (PBS), while the reagents (2-hydroxy40-(2-hydroxyethoxy)-2-methylpropiophenone) (PI) and PEGDA 98% (MW 700 Da) were used to prepare a solution at concentrations of 40% and 0.02%, respectively and vortexed for 3 min. Through polymerization performed with an Omnicure S2000 (320–500 nm, EXFO, Ontario, Canada) lamp at 100 mW/cm^2^ (measured for 365 nm) for 20 s at a distance of 16 cm, a thin layer of hydrogel was generated by adding 20 µL of the solution on top of a plate and under a cover glass. The cover glass with a thin hydrogel layer was then moved to a fresh petri dish (500 μm in diameter). A drop of hydrogel solution (500 μL) was prepared for the second layer of hydrogel. UV exposure process was performed for 27 s, and a photomask was placed on top of the cover glass. The hydrogels were washed in PBS for 24 h. DMEM medium was used to dilute HepG2 cell suspensions with an exact concentration of 0.2 × 10^6^ cells/mL. 3 mL from this solution was retrieved and added to a hydrogel microwell in each six-well plate. HepG2 spheroids were grown and maintained in an incubator at 95% air, 5% CO_2_, 37 ^o^C.

#### Drug Screening

HepG2 cells were cultured for 2 weeks to study the effects of MuA-loaded liposomes on 3D spheroids. The culture media was replaced with fresh ones on the 15^th^ day. MuA-loaded liposomes, MuA-loaded GA liposome, and blank liposome were mixed in DMEM to obtain the desired concentrations, and each 1 mL of solution was mixed with 3 mL of medium in the 6-well plate. Next, the cells were cultured at 95% air, 5% CO_2_, 37 ^o^C for 1 day. 3D HepG2 spheroids were obtained using 0.25% trypsin–EDTA for treatment, washed with PBS twice. The 0.4% trypan blue was added for staining the cells for 3 min, the stained (dead) and Unstained (viable) cells were counted on a hemacytometer, the final value was presented as % dead cells.

#### Imaging and data quantification

For the 3D reconstruction and image, an Olympus FV1000 confocal microscope was used. MetaMorph (MolecularDevice, USA) software was used for time-lapse imaging. z-projections of the images of the 3D stack images were obtained using ImageJ (NIH), which was used to quantify the vascular area coverage. The distance from the original gel and spheroid interface was the length of angiogenic sprouting and tumor cell migration.

#### Statistical Analysis

The two-way analysis of variance (ANOVA) was used for repeated measures. The Student’s *t* test was performed to analyze the data and estimate the statistical significance. For all tests, the significant difference was determined at *p* value of less than 0.05 (*P* < 0.5).

## Results

### Synthesis of GA-PEG-Chol conjugates

In this study, GA–PEG–Chol (**6**) conjugates were successfully synthesized using the routes as shown in Scheme 1. In Fig. 2A, ^1^H-NMR spectra of cholesterol (**1**) appear fully spectrum signal in the region **a** (0.8–1.3 ppm), which is a characteristic signal of CH_3_ groups, region **c** is signal of H in the group - OH and region **b** are signals of -C=CH. The intermediate Chol-suc **2** was prepared by treating cholesterol with succinic anhydric acid in DCM with the presence of DMAP by refluxing for 3 days [26]. Figure 2B is ^1^H-NMR spectra of Chol-suc (**2**). In addition to the signals of the Cholesterol framework in regions **a**, **b**, **c**, there are also signals of two groups -CH_2_ in region **d**. The resulting Chol-suc product was then purified with n-hexane/ethyl acetate (3:1), the process yield was about 65%.

Compound **2 (Chol-suc)** was then used for reaction with PEG-2000 in DCM with the presence of DCC and DMAP at room temperature for 48 h to give Chol-PEG (compound **3)**. In addition to the indications of Chol-suc (**2**) framework, signals in area **e** emerged in the ^1^H-NMR spectra of Chol-PEG (**3**) (Fig. 2C). Chol-PEG 3 product was then washed with water and dried; the yield was excellent and reached 85.4%.

Analogously, GA (compound **4**) was acetylled by acetyl chloride to give acetyl glycyrrhetinic acid (compound **5**). In Fig. 2D, the characteristic signals of GA (**4**) appear in regions **f**, **g**, **i**. ^1^H-NMR spectra of Acetyl glycyrrhetinic acid (**5**); in addition to the signals of GA framework (**4**), there is also a signal of the AcO group in the region **h** (Fig. 2E). The acetyl glycyrrhetinic acid (**5**) was rinsed with water before drying at 70 °C to obtain 78.6%, much higher than the 20% yield of process **5** which involves GTA reaction with ethylchlorofor-mate in the presence of triethylamine in dichloromethane [33].

Compound **5** was then reacted with compound **3** in DCM with mixture DCC/DMAP at room temperature for 48 h to afford the target compound GA–PEG–Chol (**6**). ^1^H-NMR spectra of GA-PEG-Chol (**6**) is shown in Fig. 2F, with signals of the GA-PEG-Chol framework appearing entirely in regions **a-i**. The signals were indicated as in Fig. 1 E. On the spectrum appear full signal of CH_3_ groups in the range of 0.8–1.7 ppm, AcO group appears at position 2.04 ppm. The dual signals at 2.60 ppm and 2.65 ppm were assigned to the cholesterol ester succinate segment, the signals at 3.45–3.78 ppm were assigned for units in PEG. The signals at 5.70 ppm were attributed to the fragment of acetylglycyrrhetinic acid. These results show that the GA-PEG-Chol conjugates have been successfully synthesized. The substance was purified by column chromatography on silica gel with dichloromethane/methanol (10:1) to yield 65% GA-PEG-Chol.

### Synthesis of liposome

The characterization of 3 types of liposomes is shown in Table 1. Three distinct liposomes with about similar sizes (around 100 nm) were obtained by using a 100 nm membrane. The product is extruded many times, thus resulting liposomes of uniform sizes without significant difference (p > 0.05). The MuA-loaded GA liposomes have size distribution indicated by PDI of around 0.139, which is significantly smaller than Blank liposomes (0.156) and MuA-loaded liposomes (0.189). In general, the PDI of all <0.2 ensures the homogeneity of liposome types. All samples are slightly negatively charged. Both MuA-loaded liposomes and MuA-loaded GA liposomes had similar Zeta potentials of −23.7 ± 1.26 and −24.2 ± 1.04 mV, respectively. The EE% and DL% are critical metrics that influence the efficiency of medication delivery systems. In the present study, MuA-loaded GA liposomes have a higher EE% and DL% (87.31 ± 2.72 and 8.13 ± 0.63, respectively) than MuA-loaded liposomes (with 83.45% ± 3.449 for EE and 7.92% ± 0.47 for DL). These findings show that the presence of GA increased the EE% and DL% of liposomes. All of the characterization assay results suggested that the prepared liposomes were eligible for further testing.

**Table 1:** Characteristics of various types of liposomes (mean ± SD)

	DLS (nm)	PDI	Zeta Potential (mV)	EE (%)	DL (%)
Blank liposomes	98.25 ± 2.68	0.156 ± 0.015	−16.38 ± 0.81	–	–
MuA—loaded liposomes	103.49 ± 4.17	0.183 ± 0.012	−23.7 ± 1.26	83.45 ± 3.49	7.92 ± 0.47
MuA—loaded GA liposomes	101.18 ± 3.26	0.139 ± 0.025	−24.2 ± 1.04	87.31 ± 2.72	8.13 ± 0.63

**Fig. 3:**
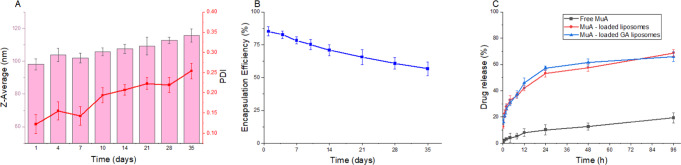
Liposome stability and in vitro drug release of MuA—loaded GA liposomes. **A** The stability of liposome in real time storage, (**B**) The change of encapsulation efficiency during 35 days, (**C**) The drug release properties of free drug and liposomes. Data are presented as the mean ± SD (*n* = 3)

The physical stability of liposomes is critical for storage, safety, and application. Liposomes are colloidal systems whose instability is characterized by particle aggregation (thermodynamic instability). Thus, the present study evaluated the thermodynamic stabilities of three types of Mu-A loaded GA liposomes by monitoring the changes in particle size and PDI to understand the drug storage behavior. Figure 3A depicts the measured size and PDI indicators at fixed time intervals in comparison to the initial. As shown in the figure, the Z-average increased slightly from about 98 nm to approximately 115 nm within 35 days. Meanwhile, over the same period, the PDI value increased sharply from 0.13 to 0.25. Figure 3B shows that the EE of MuA-loaded GA liposomes decreased with time. After 35 days, the EE gradually declined from about 85%, to approximately 63%. The opposite trend of the size and PDI compared with EE is reasonable and shows the influence of storage time on durability and sample quality.

In drug release, due to the low solubility of MuA, the release ability of free MuA over time is not significant (under 20%), while MuA-loaded liposomes and MuA-loaded GA liposomes change enormously over 35 days with 70% and around 65%, respectively (Fig. 3C). This time-varying behavior provides a more holistic view of the decision to store the drugs.

### Drug testing

The effects of blank liposome, free MuA, and MuA-loaded liposome, and MuA-loaded GA liposome on the viability of HepG2, and HUVEC cells were evaluated, at four time points: 24 h, 48 h, 72 h, and 96 h.

**Fig. 4:**
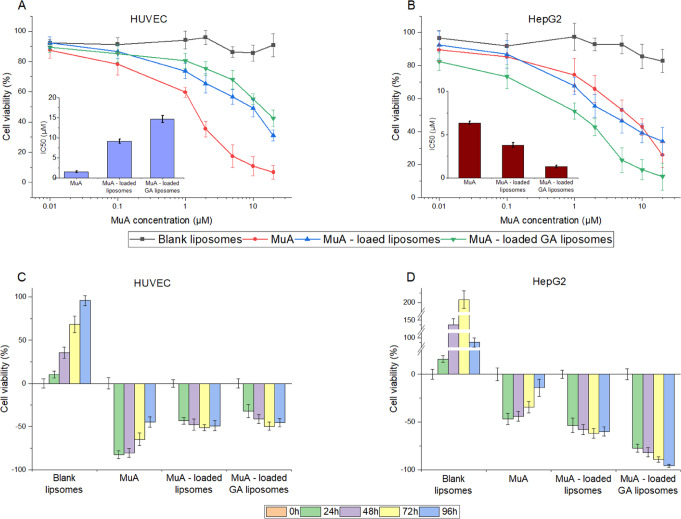
The cytotoxicity of various liposomes against HUVEC and HepG2 cells. The IC_50_ values of treatment with various formulations of MuA-loaded liposomes in HUVEC cells (**A**), HepG2 cells (**B**). Cell viability after treatment with various formulations of MuA-loaded liposomes in HUVEC cells (**C**), HepG2 cells (**D**); Data are presented as the mean ± SD (*n* = 3)

Figure 4A shows the effect of different drug concentrations on the cell viability of HUVEC. With blank liposomes, as the concentration increased, thecell viability remained almost unchanged, while the values of free MuA decreased sharply. The MuA-loaded liposomes and MuA-loaded-GA liposomes tended to decrease approximately in the same manner as the concentration of MuA increased. However, the presence of GA causes a slightly smaller decrease in cell viability. For the HUVEC cell line, the IC_50_ value of MuA is the lowest (about 2.5 µM), followed by MuA-loaded liposome (10 µM) and MuA-loaded GA liposome (15 µM). This result can be explained by the direct targeting towards cells of liposomes with high concentrations of MuA, thus causing more cell death.

Similar to the HUVEC cell line, in Fig. 4B, when increasing the concentration of MuA, the HepG2 cell viability (%) of blank liposomes almost remained, whereas this figure of free MuA decreased sharply. However, for HepG2 cells, MuA-loaded Ga liposomes caused the most cell death of all types. The IC_50_ value of HepG2 is precisely opposite to HUVEC, in which the highest value was obtained in the presence of MuA (about 7 µM); followed by MuA-loaded liposome (approximately 3.5 µM) and MuA-loaded GA liposome (around 1.5 µM). Because MuA has poor solubility, using MuA-loaded liposomes will allow the use of MuA at higher concentrations. The results also show that GA liposome has high targeting, thus it penetrates HepG2 cells better.

Figure 4C shows the cytotoxic effect of MuA and liposomes on HUVEC cells over time. The cells in the presence of blank liposome remained unchanged. In contrast, the cytotoxicity activity of MuA on HUVEC cells gradually decreased over time, in which the cell death rate reduced from 80% during the first 24 h to 50% as the treatment time reached 96 h. For the MuA-loaded GA liposomes, the cell death increased over time more strongly than MuA-loaded liposomes and reached the peak after 72 h. This result can be explained that blank liposomes allowed the cell growth, while MuA exhibited immediate killing effects on the cells. On the other hand, liposomes release drugs slowly, hence balancing the cell growth and death.

The effect of MuA and liposomes on HepG2 cells after 4 time points are illustrated in Fig. 4D. The blank liposomes for the HepG2 cell line were not affected; it remained a positive control. With the MuA, cell death decreased gradually over time, and the lowest point was only about 20% at 96 h. For MuA-loaded liposomes and MuA-loaded GA liposomes, the cell death was about 55% and 75% at the first 0 h, respectively, then increased sharply over time. The most significant increase of cell death was caused by the MuA-loaded GA liposomes, where up to 98% was killed after 96 h. This result is obtained becausethe blank liposomes were unable to inhibit the growth of cancer cells; whereas inthe presence of MuA, the cells are rapidly killed but then regenerated. GA-liposome has the ability to target and release MuA in a slow and controlled manner, thus destroying more cells. Thus, it can be concluded that among 4 tested samples, MuA-loaded GA liposomes are the most suitable for therapeutic use.

### Drug testing cell viability (2D): coculture

**Fig. 5:**
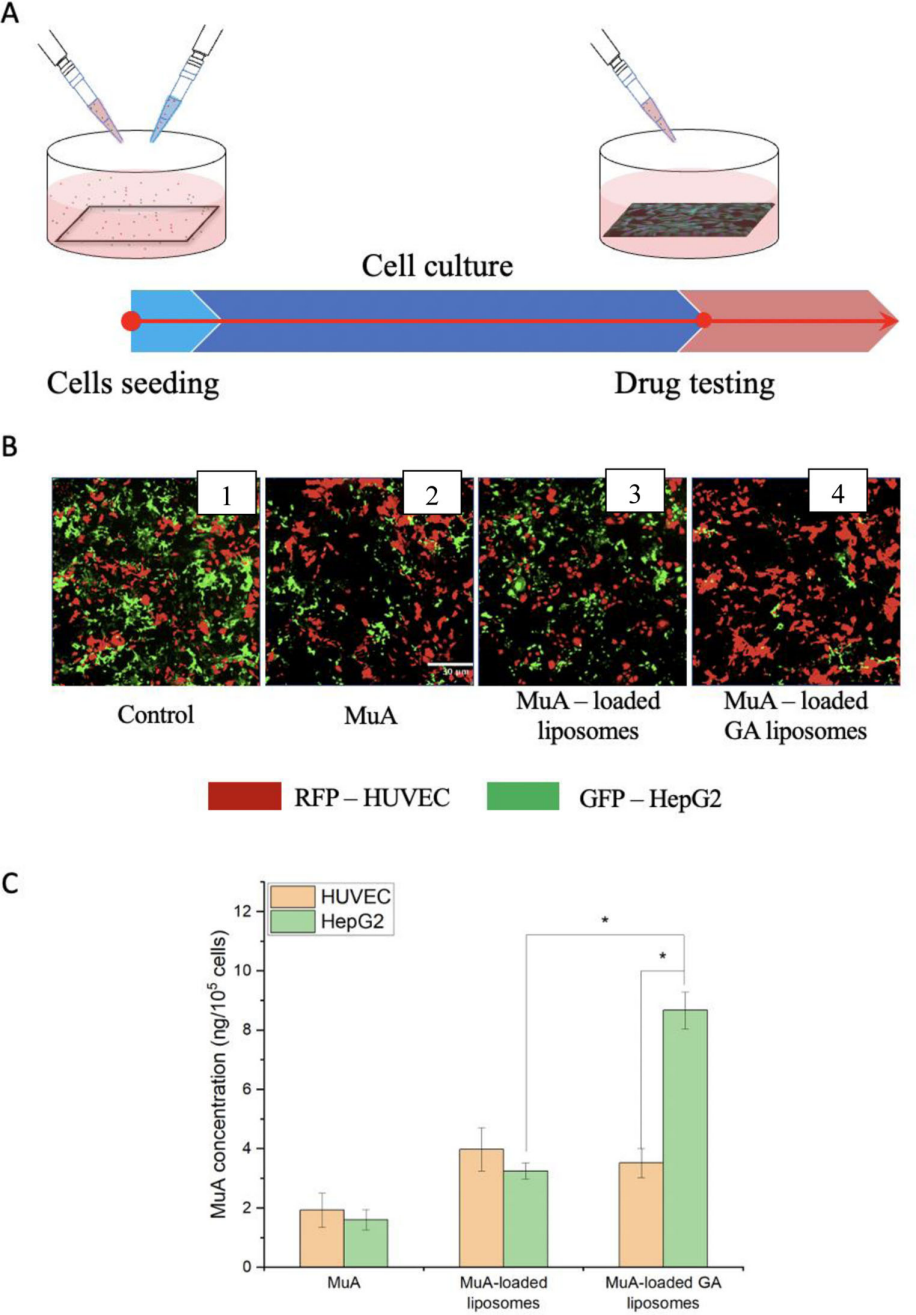
In vitro cellular uptake of various liposomes against HUVEC and HepG2 cells cocultured. **A** Schematic diagram of cocultures platform and drug testing; (**B**) Cell morphology of cocultured HUVEC and HepG2 cells before and after treatment with MuA-loaded GA liposomes. **C** Cellular uptake of HepG2 cells against various liposomes. Data are presented as the mean ± SD (*n* = 3, *p < 0.05)

Figure 5A shows a schematic diagram of the cocultures platform with HUVEC and HepG2. Two types of cell was seeded to the medium in a dish. They were cultured together at 37 ^o^C CO_2_ 5% in the incubator before drug testing.

The HUVEC and HepG2 cells before and after treatment with MuA-loaded GA liposomes are presented with the different morphology in Fig. 5B. Figure 5B-1 shows two well-developed cell types on the two sides and a conspicuous dividing line in the middle. This indicates that the control did not affect the HUVEC and HepG2 cell lines. Furthermore, MuA destroys both types of cells very quickly, with a marked reduction in the number of cells (Fig. 5B-2). Under the influence of MuA -loaded liposome, the number of cells was reduced compared to both controls because there was no specific targeting (Fig. 5B-3). In Fig. 5B-4, the number of cells on the HepG2 side is much reduced, while the HUVEC side is less changed. This result shows that MuA-loaded GA liposome mainly targets HepG2 cells.

In Fig. 5C, MuA was poorly absorbed and almost identical in both cell types (approximate 2 ng/10^5^ cells). MuA-loaded liposomes are better absorbed in HUVEC cells (4.02 ± 0.92 ng/10^5^ cells), as compared to HepG2 (3.21 ± 0.47 ng/10^5^ cells). For MuA-loaded GA liposomes, there is a marked difference, in which the absorptive capacity of HepG2 cells was up to about 8.83 ± 0.97 ng/10^5^ cells,while this capacity of HUVEC was only 3.62 ± 0.61 ng/10^5^ cells. This result is due to the presence of GA ligand in MuA-loaded GA liposome that targeted HepG2.

### Cell viability (3D): cancer spheroid

**Fig. 6:**
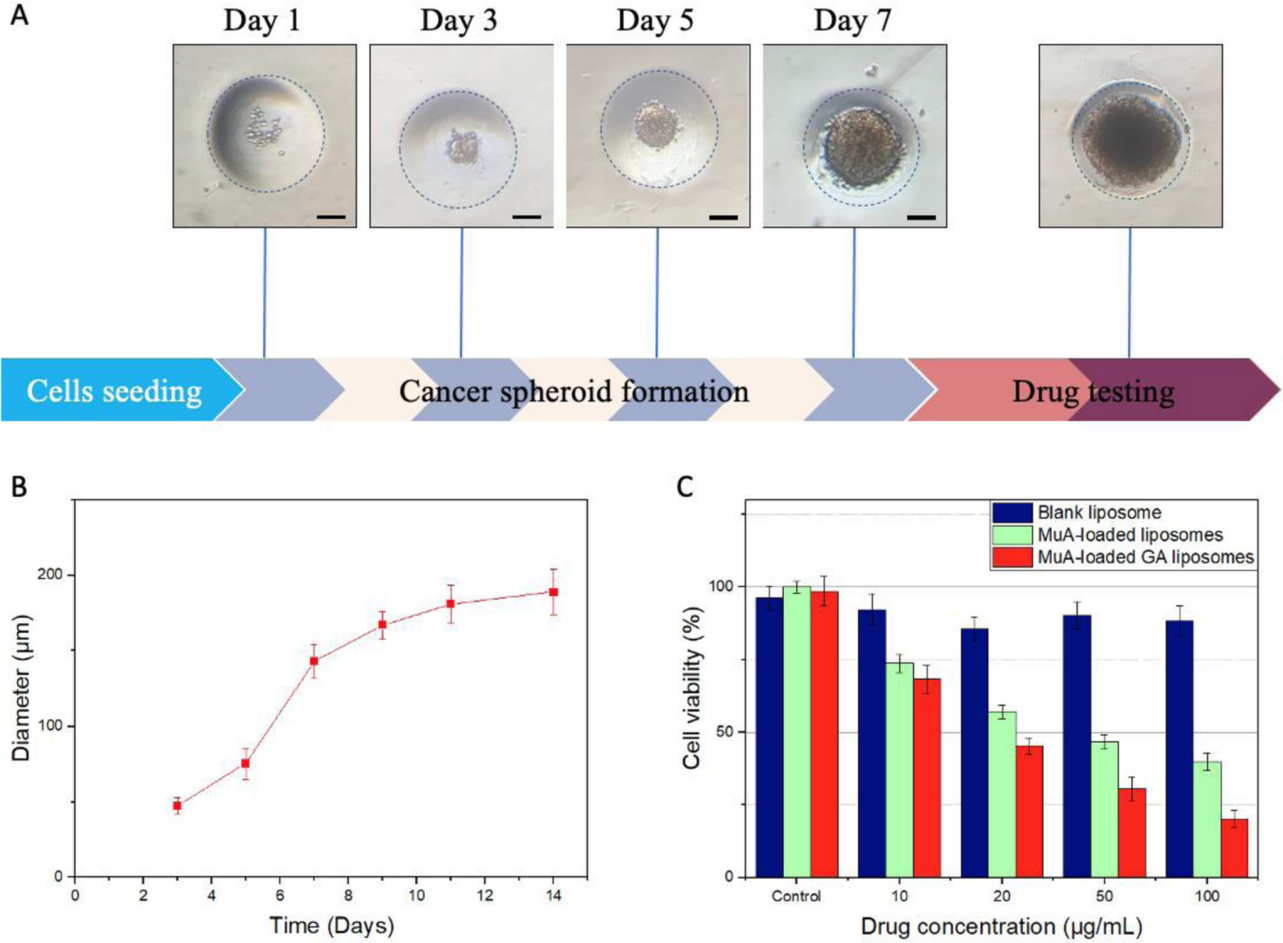
Drug testing on 3D cancer spheroid platform. **A** Timeline formation of HepG2 cancer spheroids; (**B**) increase size of cancer spheroid. **C** Cell viability of. Data are presented as the mean ± SD (*n* = 3)

Although two-dimensional (2D) cell cultures are frequently employed to assess the pharmacological effects of drugs on animal or clinical studies, they still have limitations. Thus, three-dimensional (3D) cell cultures are now being developed to recreate a model with high intensity for a better replica of the physiological conditions to accommodate better precision in drug discovery [27]. Drug testing on 3D cancer spheroid platform for HepG2 cell line was cultured for 14 days, and as the size of the cells gradually increased over time the cells were collected thoroughly for testing on the 7th dayto maintain their physiochemical nature (Fig. 6A and B). As shown in Fig. 6C, the cell viability is almost unchanged with all forms of liposomes when compared to the control. When the concentration of MuA-loaded liposomes was increased from 10 to 100 µM, cell death reduced from 100% to approximately 40%. However, high concentrations of MuA-loaded GA liposomes results in decreasing thecell viability, where the concentration of 100 µM would reduce the cell viability to only 20%. This result shows the excellent performance of MuA-loaded GA liposomes, as compared to some other synthetic liposomes which is in close agreement with previous studies where the required concentrations of co-encapsulated nano-liposomes and and bufalin-loaded PEGylated Liposomes were 500 µM and 200 µM, respectively [34, 35].

## Discussion

There are currently many methods for liver cancer therapy such as transplant, surgical resection, stereotactic body radiation therapy, and chemotherapy. However, chemotherapy with an advance of target therapy remains a synergistic treatment option [35]. Target therapy is an approach that used a molecularly targeted drug delivery systems of nanoparticles, nucleic acids, peptides, and proteins to encapsulate the small molecular drug for disease treatment [36]. Thus, it improves drug efficacy to reach therapeutic action and reduces the side effects on normal tissue. Murrayafoline A (MuA) has shown many biological activities such as anti-cell growth, anti-inflammator. By promoting the degradation of intracellular β-catenin protein, MuA attenuates the Wnt/β-catenin pathway, which is an important key in the initiation and development of hepatocellular carcinoma (HCC), cholangiocarcinoma (CCA), and hepatoblastoma (HB) [37]. However, MuA has poor aqueous solubility which can limit the dose usage of MuA in cancer treatment. Moreover, MuA has high cytotoxicity so it can accidentally destroy other normal cells, such as bone marrow, hair follicles, and epithelial cells in the mouth, digestive tract, and reproductive system. To reduce the negative effects of MuA, several drug delivery methods have been developed and applied to solve these problems [37]. For example, Xie et al. used riple-stimuli-responsive nanocarrier based on graft copolymer assembly to store and delivery anti-cancer drug to increase circulation time, and control intracellular drug release. However the complexity of such nanocarriers increases dramatically and work is still required to demonstrate the clinical efficacy of these systems. Another study from Gu *et al*. used enzyme-responsive nanocarriers to exhibite good colloidal stability under physiological conditions and high drug loading content but these methods are still required to fully develop such systems [38]. In this study, liposomes were used for drug delivery and showed that the particle size was as small as 100 nm, the encapsulation efficiency and drug release increased rapidly of up to 60% and increase stablely after 48 h.

To enhance the effect of targeting the liver, GA was used as ligands to modify liposomes to specifically target malignant liver cancer. Our results in coculture experiment showed that MuA-loaded GA liposomes absorptive capacity of HepG2 cells was up to about 8.83 ± 0.97 ng/10^5^ cells, almost 2.5-fold higher than that of HUVEC cells. Compared with Yan’s results, ligand GA in the outermost has a directional for liposomes to target GA receptors, which have been found to be 1.5- to 5-fold in cellular membrane of hepatocytes more than normal cell [39]. Yang et al. used a polymer micelle carrier in combination with GA as the target vector to inhibit the proliferation of HepG2 cells. In this experiment, drug accumulation in HepG2 tumor-bearing mice treated with the GA modified micelles was higher than drug solution after 24 h [40]. In addition, the study of Tian *et al*. using modified nanoparticles such as chitosan-modified GA also showed that the accumulation in the liver was significantly higher than in other tissues [41]. GA was attached to modified sulfated chitosan micelle, however its EE results ranged from 55.81% to 72.74% and when improve the hydrophobicity of carriers, the EE increase significantly [41].

In Jia et al. study, the water insolubility of GA was enhanced by using PEG-modified liposomes. The results showed that GA-PEG modified liposomes to increase the packing efficiency, stability and accumulation in cells than GA modified liposomes [42]. In this study, GA is attached to Chol – a stalibized proporation of liposome across the PEG. Thus, GA binds to Chol to minimize the change of liposome properties. Moreover, PEG groups could surround the liposomes to minimize recognition and elimination when liposomes move through the body. It also forms an outer shell that can reduce liposome aggregation, save the system stability and prolong the circulation time. Having an OH group in the molecule, it is easier to attach PEG and GA to Chol than with phospholipids. The reaction process as shown in Fig. 1 to produce the final product is GA-PEG-Chol. The substance was purified and yield 65% GA-PEG-Chol.

Liposomes were synthesized by the film hydration method followed by extrusion. In order to modify liposomes with GA-PEC-Chol, the GA-PEG-Chol conjugates were synthesized from cholesterol, PEG-2000, and glycyrrhetinic acid in 3 displayed steps in Scheme 1. Cholesterol was mixed with anhydrite succinic to create Chol-suc. After purification, Chol-suc was added to DCM and DCC with PEG 2000 for synthesis PEG-Chol, and the molar ratio of the mixture is 7:4. The compound of PEG-Chol was formed and acts as a stabilizing component for the structure of liposomes. This compound has been combined in a 1:1 molar ratio with GA, which helps the liposomes can precisely target the liver. Finally, the GA-PEC-Chol can be mixed into the high DOPC proportion, which was verified by the results of Section 3.1 and Section 3.2.

The aqueous core of the liposomes was applied to encapsulate an anti-HCC agent - MuA. In Table 1, all liposomes exhibited an even size distribution ranging from 96 to 107 nm (<200 nm) with the low relative polydispersity index. With small size, liposomes can intrusion and accumulate in cancer cell more easily than other carriers, owing to their enhanced permeability and retention (EPR) effects [43, 44]. Moreover, the zeta potential value of three types of liposomes have a high negative charge from –16.38 mV (blank liposomes) to 24.2 mV (MuA-loaded GA liposomes), which supported more profitably for stabilizing in aqueous solution. Previous studies have shown that during drug delivery, the highly positively charged system suffers from decreased stability in blood because it tends to interact with plasma proteins. Meanwhile, with a low negative charge drug delivery system, stability in blood is enhanced and it can encourage cellular uptake [42]. In *Jia* et al. study, GA was attached to PEG-modified liposomes, and the modified liposomes have size distribution with diameters near 120–160 nm and the zeta potential is 22.52 V mV [42].

To test the effect of GA-liposome, two cell types, HUVEC - primary cells isolated from the vein of the umbilical cord and HepG2- human hepatoma cell line, were used. HUVEC was used for co-culture with HepG2 because HUVEC is one of the most susceptible blood vessel-forming factors to cancer therapeutic effects, and it is normally close to the cancer area. As shown in Fig. 4A, free MuA is better at killing HUVECs due to its high cytotoxicity, while MuA-loaded liposomes and MuA-loaded GA liposomes need time to release MuA to take effect on cells. However, compared with HepG2 liver cancer cells in Fig. 4B, with GA directing liposomes to HepG2 cells, the cell viability of MuA-loaded GA-liposomes was lower. In addition, because the proliferation rate of cancer cells is stronger and faster than that of normal cells, the cytotoxicity results of MuA (Fig. 4C, D) are different between HUVEC and HepG2 cells lines. However, the release behaviors of MuA-loaded liposomes and MuA-loaded GA liposomes were in a different trend with free MuA that HepG2 has lower cell viability than HUVEC. The most obvious difference in viability was the MuA-loaded GA liposome with the main reason for this was the presence of abundant GA receptors on hepatocyte membranes [41].

To further explore the targeting effect of GA ligand-modified liposomes, HUVEC and HepG2, which present with different receptors, were used and cocultured. Remarkably, the cell density was strongly reduced after two cell types were treated with MuA, MuA-loaded liposomes, and MuA-loaded GA liposomes. From Fig. 5B-4, it can be seen that when using the GA ligand as a target to the hepatocytes, the density of HepG2 decreased significantly. Moreover, in the Fig. 5C, when MuA was encapsulated in two types of liposomes, the cellular uptake was markedly increased for liposomes modified with GA. In addition, MuA uptake by HepG2 cells increased approximately 2,6-fold compared with HUVEC cells. The results suggest that the presence of GAs in liposomes could prompt high MuA concentrations in the cells by specifically targeting hepatocytes and achieving notable cytotoxicity [45]. Therefore, it was concluded that GA liposomes have a higher affinity for HepG2 cells than free liposomes.

Although two-dimensional (2D) cell cultures have been used to evaluate the pharmacological effects of MuA on cells with promising results, three-dimensional (3D) cell cultures were performed to replicate better than physiological conditions to meet better accuracy in drug discovery [27]. Figure 6 shows that, after 14 days of culture, HepG2 cells develop normally and the size of spheroids is approximately 200 µm. Due to the small size of the drug delivery system, MuA-loaded liposomes and MuA-loaded GA liposomes assisted MuA to enter the cancer spheroids and reduce the cell viability of HepG2 when MuA concentration was increased. The weakest viability in cells was observed with MuA-loaded GA liposomes. With the precise targeting of the GA to the GA receptor on the hepatocyte cell membrane, MuA can achieve significant cytotoxicity to kill cancer cells. Comparing the results with *Jia et al*. study, GA-PEG-modified liposomes target and induce cytotoxicity against liver cancer cell lines better than free drug and drug-loaded non-modified liposomes. It can accumulate in tumors 48 h after treatment and inhibit tumor growth in mice by more than 90% after 2 weeks [42].

## Conclusion

This research has successfully synthesized GA-PEG-Chol conjugates, which contain the liver-targeted substances, and successfully modified the liposomes of MuA. The synthesized drug efficiency and safety was tested in vitro on 2D and 3D cell cultures of HUVEC and HepG2 cancer cell lines. The results showed that MuA-loaded GA liposomes displayed higher efficiency than MuA and MuA-loaded liposomes drugs. The IC_50_ values of MuA-loaded GA liposomes Tested on 2D cell culture of HepG2 and HUVEC cell line were 2 µM and 15 µM, respectively. The cellular uptake of MuA-loaded liposomes in HepG2 and HUVEC cells co-culture was 8.83 ± 0.97 ng/10^5^ cells and 3.62 ± 0.61 ng/10^5^ cells, respectively. Upon the drug testing on 3D cancer spheroid platform of HepG2 cancer spheroids, MuA-loaded GA liposomes had the highest efficiency at a concentration of 100 µg/mL. The above results indicated that MuA-loaded GA liposomes had significant liver targeting activity, as compared to MuA-loaded liposomes, and GA is a suitable ligand for sustainabletargeted drug delivery for liver cancer treatments.
